# Sarcopenia Diagnosis and Management in Hematological Malignancies and Differences with Cachexia and Frailty

**DOI:** 10.3390/cancers15184600

**Published:** 2023-09-16

**Authors:** Giuseppe Ferdinando Colloca, Andrea Bellieni, Beatrice Di Capua, Marialuisa Iervolino, Serena Bracci, Domenico Fusco, Luca Tagliaferri, Francesco Landi, Vincenzo Valentini

**Affiliations:** 1 Dipartimento di Diagnostica per Immagini, Radioterapia Oncologica ed Ematologia, Fondazione Policlinico Universitario A. Gemelli IRCCS, 00168 Rome, Italy; giuseppeferdinando.colloca@policlinicogemelli.it (G.F.C.); serena.bracci@guest.policlinicogemelli.it (S.B.); vincenzo.valentini@policlinicogemelli.it (V.V.); 2 Centro di Eccellenza Oncologia Radioterapica e Medica e Radioterapia, Ospedale Fatebenefratelli Isola Tiberina—Gemelli Isola, 00186 Rome, Italy; 3 Dipartimento Universitario di Scienze Geriatriche ed Ortopediche, Università Cattolica del Sacro Cuore, 00168 Roma, Italy; 4 Dipartimento di Scienze dell’Invecchiamento, Ortopediche e Reumatologiche, Fondazione Policlinico Universitario A. Gemelli IRCCS, 00168 Roma, Italy; domenico.fusco@policlinicogemelli.it (D.F.); francesco.landi@unicatt.it (F.L.)

**Keywords:** sarcopenia, geriatric oncology, frailty, hematology

## Abstract

**Simple Summary:**

Sarcopenia is a geriatric syndrome characterized by progressive and generalized loss of muscle strength and muscle mass. It associated with reduced physical function and negative outcomes such as falls, hospitalization, loss of autonomy, disability, and mortality. Sarcopenia has bought value in the cancer management, given its impact on patients’ treatments and prognosis. In hematologic diseases, sarcopenia predicts toxicity, treatment’s response, influences overall survival and non-cancer-related risk of death. Diagnosing and properly framing sarcopenia is of great importance in designing the patient appropriate treatment and to perform a personalized or “tailor made” approach and supportive care. Early recognize sarcopenia allows to reverse the muscle loss process and to avoid negative impacts of sarcopenia syndrome on the patients’ trajectory.

**Abstract:**

Sarcopenia is a geriatric syndrome characterized by a progressive loss of systemic muscle mass and decreased muscle strength or physical function. Several conditions have a role in its pathogenesis, significantly impacting adverse outcomes such as falls, functional decline, frailty, disability, multiple hospitalizations, and mortality. In the oncological setting, sarcopenia is associated with an increased risk of treatment toxicity, postoperative complications, and a higher mortality rate related to other causes (e.g., pneumonia). In the hematological field, even more so, sarcopenia predicts toxicity and response to treatments. In patients with hematologic malignancy, low muscle mass is associated with adverse outcomes and is a predictor of overall survival and non-relapse mortality. Therefore, it is essential to correctly recognize sarcopenia, evaluate the risk factors and their impact on the patient’s trajectory, and effectively treat sarcopenia. Sarcopenia is a reversible condition. The most effective intervention for reversing it is physical exercise combined with nutrition. The objective of clinical assessment focused on sarcopenia is to be able to carry out a “tailor-made treatment”.

## 1. Introduction

From the etymological to the functional, numerous definitions of sarcopenia have been developed over time, completely remodeling its original meaning. Currently, sarcopenia is one of the most studied and researched pathological syndromes in the geriatric field, as its presence correlates with adverse outcomes. It is crucial in non-geriatric settings to learn how to recognize sarcopenia, given its impact on cancer patient management.

Sarcopenia is one of the leading causes of frailty in older adults, so much so that according to some authors, the two concepts should be merged, or sarcopenia should be considered one of the causes of the frail condition [1].

The term comes from the Greek words “sarx” (flesh) and “penia” (loss). First identified by Rosenberg in 1989 [2], sarcopenia was considered an age-related decline in lean body mass affecting mobility, nutritional status, and independence.

Currently, from the historical definition of sarcopenia as an idiopathic loss of muscle mass, we have moved on to a functional definition in which qualitative and quantitative alterations of muscle function impact the person’s ability to resist stress, consequently making him more exposed to adverse outcomes, increased risk of being institutionalized, and increased risk of death. This framework is the focus of current geriatric research [1,2,3,4,5,6].

The high predictiveness of adverse outcomes and the increased risk of toxicity to treatments meant that sarcopenia quickly became a topic that moved from the geriatric scenario to the oncological one. The sarcopenic condition is not associated with the risk of cancer but rather with the risk of treatment-related toxicity and, consequently, the possibility of not completing treatments or not having access to them [7,8,9,10,11,12].

Oncological and hematological treatments are associated with a reduction in physical activity (due to hospitalization, numerous check-ups, and treatments that often have to be carried out in distant centers, such as radiotherapy) and variations in caloric and nutritional intake (i.e., for nausea, inappetence, or dietary indications related to specific treatments). The two conditions: age and oncological therapies, can determine a state of pre-sarcopenia, which can evolve up to sarcopenia [13].

The current definition of sarcopenia comes from gerontology research on longevity and the mechanisms underlying successful aging and frailty. Ferrucci had identified muscle strength as a factor related to aging processes [14]. Goodpaster highlighted how, with physiological aging, even healthy individuals showed a progressive reduction in muscle mass, even while maintaining a stable weight [15]. This phenomenon is linked to an advanced process of fatty infiltration of the muscle, with performance loss while maintaining the same muscle mass. In Goodpaster’s studies, only individuals with an increase in body weight had an increase in muscle mass, but this increase in mass did not correspond to an improvement in performance [16]. Based on these two aspects, the definition of sarcopenia has evolved from a simple progressive reduction of muscle mass to a qualitative alteration of lean muscle mass related to performance. Thus, measuring muscle performance (strength) is essential for a sarcopenia diagnosis. The current definition considers sarcopenia a progressive skeletal muscle disorder associated with an increased likelihood of adverse outcomes [16]. The mentioned aspects make the differential diagnosis of malnutrition, eating disorders, and cachexia necessary.

In the once-hematological scenario, Prado was the first to identify a relationship between sarcopenia and treatment-related toxicity [17]. Zilioli highlighted an increased risk of recurrence in the sarcopenic older patient with non-Hodgkin’s lymphoma (NHL) [18,19]. In this manuscript, the aims are to highlight those aspects useful in the assessment of an older cancer patient affected by hematologic disease to identify sarcopenia, prevent treatment-related toxicities related to the presence of sarcopenia, and improve the patient’s quality of life through the prevention and treatment of sarcopenia.

## 2. Definitions and Features: Sarcopenia, Cachexia, Frailty

### 2.1. Sarcopenia

Sarcopenia is a geriatric syndrome characterized by a progressive and generalized skeletal muscle disorder that involves the accelerated loss of lean muscle mass and loss of function, closely related to aging mechanisms. It is associated with increased adverse outcomes, including falls, functional decline, frailty, disability, multiple hospitalizations, and mortality [20,21,22]. In 2010, the most widely cited definition was proposed by the European Working Group on Sarcopenia in Older People (EWGSOP) [23], in which the concept of muscle function was introduced in the consensus definition. Muscle function, defined by muscle strength, power, or physical performance, has been considered because function was consistently a more powerful predictor of clinically relevant outcomes than muscle mass alone [24,25].

**Figure 1:**
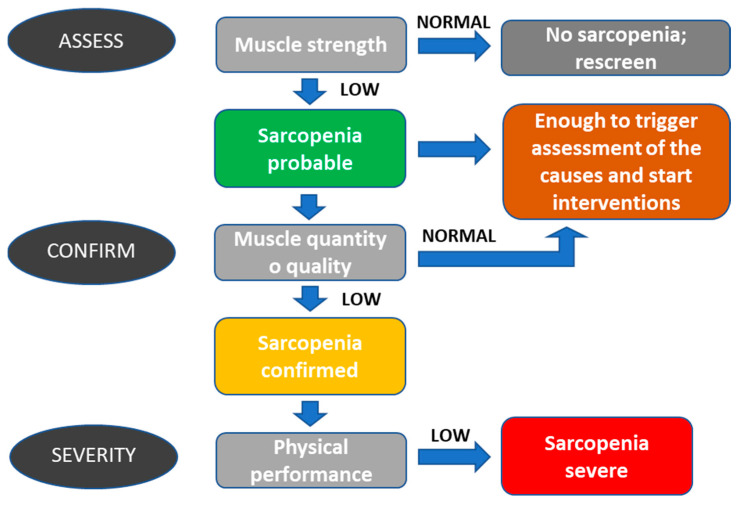
Modified sarcopenia EWGSOP2 algorithm for case finding, making a diagnosis, and quantifying severity in practice [26]. Diagnosis starts with a measure of muscle strength to suspect sarcopenia (sarcopenia “probable”). The second step is the measurement of muscle mass in quantity and quality (sarcopenia confirmed). Even in the case of probable sarcopenia, we suggest starting the assessment of causes and interventions to prevent further deterioration. It is essential to rescreen the patients regularly because a decrease in muscle strength can appear anytime.

The operational definition of sarcopenia suggests a conceptual staging as “presarcopenia,” “sarcopenia”, and “severe sarcopenia” [Figure 1]:

The “sarcopenia” stage is characterized by low muscle mass, strength, or physical performance. “Severe sarcopenia” is identified when all three definition criteria are met (low muscle mass, low muscle strength, and low physical performance) [26] [Table 1].

Lastly, in 2016, the International Classification of Diseases-10 code recognized sarcopenia as an independent condition.

The pathogenesis of sarcopenia is multifactorial. The condition is related to a variety of chronic diseases, including chronic obstructive pulmonary disease (COPD), chronic kidney disease (CKD), cardiovascular and cerebrovascular conditions, metabolic and endocrine disorders [27,28,29], and risk factors including age, gender, physical activity level, disuse, malnutrition, and cachexia [30].

Sarcopenia can be considered severe when low muscle strength, low muscle quantity/quality, and low physical performance are detected.

### 2.2. Sarcopenic Obesity

The progressive population aging associated with age-related physiological changes (reduction of lean muscle mass and increase in fat mass) and the increasing prevalence of obesity observed in recent decades has led to the development of a new pathological condition defined as sarcopenic obesity (SO), characterized by a mismatch between muscle mass and fat mass. The prevalence of SO increases with age from 10% to 15% [31,32,33]. 

Evidence shows that SO is associated with morbidity and a higher risk of physical frailty. In any healthy individual, bone and muscle grow harmoniously with weight change. This harmony is maintained by gravity stimulating the mechanoreceptors in bone and muscle that modulate the production of growth factors [34]. This adaptive physiological mechanism may be impaired in some older individuals who become obese without a parallel growth of muscle mass and strength. According to the World Health Organization [35], obesity is defined as body mass index (BMI) ≥ 30 kg/m^2^, and central obesity as a waist circumference greater than 102 cm in men and 88 cm in women. In OS, obesity is associated with an age-associated decline of muscle strength and function, mainly due to a parallel decrease in muscle mass. OS is not found in all obese subjects but only in those whose fat mass increase does not match lean mass and strength growth. The progressive deterioration of muscle “quality” includes a decrease in size and the number of fibers, intrinsic contractility reduction in the intact fibers, fat micro- and macro-infiltration, collagen increase, motor unit modification, and impaired neurological modulation of contraction [36,37,38].

### 2.3. Frailty

Sarcopenia is usually associated, in the geriatric field, with frailty. Both because it is considered by some to be a geriatric syndrome and because it is seen as the basis of the risk of frailty. The frailty condition is a syndrome characterized by physical, cognitive, and socioeconomic performance aspects. The exact operational definition of physical frailty (fragile phenotype) identified by L. Fried today includes the parameters used to identify it [23].

Frailty has been defined as a state of vulnerability to poor resolution of homeostasis after a stressor event due to the cumulative decline in many physiological systems [39]. 

### 2.4. Cachexia

Sarcopenia and cachexia are often confused as they have similar operational definitions, especially considering muscle mass. However, there are numerous differences between the two conditions, mainly represented by the possibility of reversion in the case of sarcopenia and the condition of sarcopenic obesity, which makes a diagnosis of sarcopenia plausible even in overweight or obese patients. The latter state cannot be achieved in the case of cachexia [1]. Recognizing and distinguishing these two conditions, particularly the stages of sarcopenia, may help select treatments and set appropriate recovery goals [40] (Table 2).

The term cachexia is derived from the Greek words “kakòs” (bad) and “hé́xis” (condition). It may be defined as a multifactorial syndrome characterized by severe body weight, fat, and muscle loss, and increased protein catabolism due to underlying disease(s). Cachexia is clinically relevant since it improves patients’ morbidity and mortality. Contributory factors to the onset of cachexia are anorexia and metabolic alterations, i.e., increased inflammatory status, increased muscle proteolysis, and impaired carbohydrate, protein, and lipid metabolism [1,41].

The biological substrates of sarcopenia and cachexia largely overlap, although some specific pathways are observed in sarcopenia but not in cachexia, and vice versa. The decrease in muscle mass is a standard feature, while weight loss and fat mass reduction are commonly seen in cachexia but not typically found in sarcopenia. In cachexia, we can always find the specific disease that led to this condition, whereas sarcopenia is a multifactorial condition that can often occur without a triggering illness [42]. 

Evans et al. proposed specific criteria for diagnosing cachexia: weight loss of at least 5% in 12 months or BMI < 20 kg/m^2^, associated with three or more of the following criteria: muscle weakness, fatigue, anorexia, low skeletal muscle index, and abnormal biochemistry (increased inflammatory markers, anemia, low serum albumin) [41]. The international consensus on cancer cachexia describes cachexia as a continuum that can develop progressively through three levels of severity: pre-cachexia, cachexia, and refractory cachexia. However, not all patients traverse the entire spectrum [43].

## 3. Causes of Sarcopenia

Several factors cause sarcopenia. The risk factors include age, gender, low physical activity, and chronic disease [44,45] [Table 3].

Aging perturbs the homeostasis of skeletal muscle: the modification in the levels of growth hormone (GH), testosterone, thyroxine, and insulin growth factor (IGF) negatively affects muscle quality and strength [46], in particular reducing protein synthesis and metabolism of skeletal muscle cells [47].

Cellular changes in sarcopenic muscle include reduction of myofibers and quality with intramuscular and intermuscular fat infiltration [48,49,50].

Molecular changes involve alterations to complex signaling pathways, including IGF-1, box protein transcription factor, and other interlinked pathways [51].

Chronic low-grade inflammation contributes to a loss of muscle mass, strength, and function; therefore, high levels of cytokines like IL-8 and IL-2R are often elevated in sarcopenic patients [42,46].

### 3.1. Inflammation

The pro-inflammatory state may be one of the critical factors in creating a vicious cycle of decreased muscle strength among obese people. Adipose tissue is an active metabolic tissue that produces pro-inflammatory cytokines, such as interleukin (IL)-6 and tumor necrosis factor (TNF)-α, and adipokines, such as leptin and adiponectin, that up-regulate the inflammatory response [52]. Pro-inflammatory cytokines were positively related to fat mass and negatively to muscle mass [53]. The InCHIANTI study found that obese community-dwelling older persons with low muscle strength had elevated levels of CRP and IL-6 compared to those with standard strength [54]. Insulin is an anabolic signal for proteins. Inflammatory molecules mediate obesity-related insulin resistance, may promote muscle catabolism, and are an independent correlate of poor muscle strength. On the other hand, resistance training improves insulin sensitivity and glycemic control [55]. Thus, low muscle strength and obesity may be connected, making them more likely to be associated than expected by chance alone.

### 3.2. Malnutrition and Weight Loss

Older adults tend to have too little protein intake in their diet, which impairs protein muscle turnover, especially during weight loss, such as during cancer treatment; this scenario is often related to accelerated sarcopenia [56].

Malnutrition, an imbalance between energy, protein intake, and other nutrients, can aggravate the decline in muscle function and quality [57]. It’s prevalent in older adults due to several factors, like lower levels of physical activity and a reduction in energy needs, decreased appetite, chewing problems, poor digestion and absorption function, and cognitive decline [58].

## 4. Management of Sarcopenia

The potential for treating sarcopenia with pharmacological and nonpharmacological interventions has been the subject of several clinical trials and meta-analyses. However, at present, the evidence on the effectiveness of these interventions is only partially conclusive. Physical exercise is a crucial weapon against sarcopenia, with greater significance when combined with a specific dietary regimen or nutritional supplements [1]. In contrast, pharmacological interventions are not an adequate alternative to date regarding the balance between efficacy and tolerance [1]. Evidence of the effectiveness of physical exercise and nutrition comes from clinical trials conducted on sarcopenic community-dwelling elderly. At the same time, literature on cancer patients, particularly former hematological patients, is lacking. 

Concerning physical exercise, the scientific community agrees with the effectiveness of a multimodal intervention in sarcopenic patients, including resistance training, walking, aerobic training, and balance training. Resistance exercise should involve all major muscle groups with a total-body approach [59]. Such physical activity, carried on for at least 6 to 12 weeks, has effectively increased muscle mass and strength in sarcopenic patients, including patients with sarcopenic obesity [60,61,62].

Regarding nutrition, the general recommendations for optimal intake of macro- and micronutrients for sarcopenia are 24–36 kcal per kg of body weight and 1.2–1.5 gr per kg of protein, distributed over the three main meals, while ensuring vitamin D intake is sufficient to maintain adequate serum blood levels (100 nmol/L) and proper supplementation of antioxidants, amino acids, and omega-3 fatty acids [63,64,65,66]. The higher protein intake is due to the slower anabolism of the elderly muscle, which should be adequately stimulated [67,68,69]. 

It is also essential to consider that animal-derived proteins stimulate muscle anabolism more than plant-derived proteins. Thus, the intake should be even higher whenever a significant component of plant-derived proteins is included in the diet [70].

Another factor to consider is fat intake. Recent evidence shows that patients who consume high amounts of fat are at risk of sarcopenia even if the expected protein intake is adequate according to the recommendations [70]. In cases of malnutrition, oral protein supplements should be considered [71].

What is clear about nutrition and exercise is that combining the two leads to the best results and is always desirable [61,72]. 

As mentioned above, no specific drugs have been approved for treating sarcopenia, as no compound tested to date has shown efficacy and good tolerance in the older population [73].

## 5. Cancer Treatments and Sarcopenia

Sarcopenia is being consistently recognized as a condition not only associated with the presence of malignancy but also induced by oncologic therapies. Due to its negative impact on tolerance to chemotherapy and outcomes in both medical and surgical cancer patients, sarcopenia should always be considered, prevented, and, if recognized, appropriately treated. Sarcopenia and cancer treatments have multiple interconnections.

On the one hand, chemotherapy can interfere with muscle metabolism, which can be associated with the onset or worsening of sarcopenia. The progressive erosion of muscle mass during oncologic therapy might be partially attributed to uncontrolled muscle protein catabolism, which intensifies as tumor growth progresses [74,75].

Some authors [76] emphasize that adverse events during chemotherapy, such as fatigue, loss of appetite, nausea, vomiting, and diarrhea, can negatively affect food intake and physical activity and contribute to an aggressive loss of muscle mass. 

Mechanisms responsible for an alteration of muscle metabolism are not fully elucidated. Some molecular pathways, however, have been recognized.

The autophagy–lysosome pathway (ALP) targets eliminating mitochondria and other cellular components. The ubiquitin–proteasome pathway (UPP) is involved in the degradation of myofibrillar proteins. Insulin-like growth factor 1 (IGF-1) and others may interfere with the synthetic processes.

Some authors have proposed a link between gains in muscle mass and stable disease [77]. Patients with significant improvements in muscle mass showed a better response to treatment, could eat well, and had reasonable symptom control. In contrast, those who lost muscle mass had rapid disease progression and short survival.

Body composition is an essential feature in cancer patients as it may affect the efficacy and toxicity of chemotherapy and is associated with patient outcomes in terms of functional status, surgical complication rates, length of hospital stay (LOS), and overall survival (OS) [78]. Differences in body composition may underlie some of the heterogeneity in chemotherapy toxicity by altering chemotherapy distribution, metabolism, and clearance. In aging, loss of skeletal muscle and an increase in adiposity result in a lower lean body mass relative to overall body weight, thus potentially causing a lower volume of distribution and higher drug concentrations [79].

Anticancer drugs display various pharmacokinetic (PK) properties. Sarcopenia is a predictive factor of anticancer drug toxicity and may be more relevant for drug dose calculation than the “classical” body surface area or flat-fixed dosing [80,81].

The pharmacokinetics hypothesis assumes that toxicity may be explained by overexposure to anticancer drugs in sarcopenic patients due to changes in tissue relative proportion between fat and lean body mass. From a pharmacological perspective, sarcopenia increases drug exposure, and several studies showed a correlation between lean body mass, drug exposure, and toxicity [7,82,83].

Another explanation for overexposure in sarcopenic patients may be decreased activity of liver cytochromes (CYP) that are involved in the metabolism of numerous anticancer drugs. Besides cytochromes, other enzymes are involved in drug metabolism and may be modulated by nutritional status and cancer-related sarcopenia. In addition, by reducing liver enzyme activity, sarcopenia is associated with a lower clearance for drugs whose elimination relies on liver metabolism (characterized by a low extraction ratio), including anthracyclines [84]. However, data on changes in pharmacokinetics with age are conflicting [85].

Alternatively, the pharmacodynamics hypothesis postulates that, independently from PK, sarcopenic patients are more sensitive to treatment and may experience toxicities even without an overdose. This is related to the concept of frailty, which has become increasingly recognized as one of the most critical issues in health outcomes and is of particular importance in cancer patients [86].

## 6. Sarcopenia in Hematological Patients

Sarcopenia is considered a predictor of disease outcome in several malignant diseases. DLBCL is the most prevalent non-Hodgkin lymphoma and is regarded as an aging-related disease [81].

A meta-analysis about the role of sarcopenia in patients with malignant hematological diseases showed that sarcopenia is associated with lower overall survival in patients with diffuse large B cell lymphoma (DLBCL) treated with modern chemotherapy [81].

Estimating muscle loss doesn’t require additional investigations and costs because most radiological tests for sarcopenia are based on computerized tomographic scans, which are also used for staging lymphomas. Therefore, an estimation of sarcopenia on producing CT should be performed, and the results should be provided to oncologists to optimize therapy and clinical outcomes [79].

The evolutionary course of older adults diagnosed with DLBCL may be modified thanks to individualized treatment allocation and functional tailoring based on a CGA that includes specific geriatric performance assessment tools [31,32,87].

The implementation of rehabilitation measures, such as multicomponent exercise programs [49], when functional decline is identified must be a core component of managing adults with cancer [50]. Even in non-sarcopenic patients, physical exercise prescription to preserve functional reserve and avoid the onset of sarcopenia after the diagnosis is an important topic. As shown in the study by Garcia Baztan et al., non-sarcopenic patients at baseline could experience a functional decline during the treatment protocol. These findings reinforce the importance of evaluating performance status after diagnosis, and they warn about functional decline as a CT non-toxic adverse event that may increase the risk of physical dependence in cancer survivors [47].

Although several specific hematologic assessment tools have been developed for the initial assessment of patients with DLBCL in settings with low availability of geriatricians [10], most guidelines recommend implementing a comprehensive geriatric assessment (CGA) for toxicity risk evaluation in older patients.

There are no reports about the relationship between sarcopenia and OS in other frequent and less frequent hematological malignancies like acute and chronic lymphatic leukemias, follicular cell lymphoma, mucosa-associated lymphoma, marginal zone cell lymphoma, and Burkitt lymphoma [81].

## 7. Conclusions

Muscle strength is more important than mass as a determinant of functional limitation and poor health in older adults. Low muscle strength has proven to strongly predict functional capacity, institutionalization, and mortality. Evidence indicates that obesity and muscle impairment can co-exist, acting synergistically at the risk of developing multiple health-related outcomes. Sarcopenic obese individuals are more likely to be disabled than individuals who are just obese or sarcopenic.

Sarcopenia represents a new scenario in the medical landscape to be evaluated and recognized as correlated with adverse outcomes. In the hematological field, even more so, sarcopenia predicts toxicity and response to treatments.

The key aspects can be represented as follows:

- Identification of sarcopenia. This process must be quick and easy to use. The tests chosen for a screening or diagnostic evaluation should be easily feasible. Therefore, the one that can be reasonably and quickly performed on all patients in the context of the reference structure must be chosen among the available tests.
- Evaluation of sarcopenia as an influencing factor on toxicity and response to treatment. It is essential to discuss treatment options to reduce the risk of toxicity to the patient and start treating sarcopenia or, in any case, reduce its impact on the patient.
- Treatment of sarcopenia. All sarcopenia treatments are directed towards an improvement in the quality level of the muscle. This is obtained by increasing protein intake, which is associated with increased aerobic and counter-resistance physical exercise. It is essential to remember that muscle mass is a complex parameter to monitor as much as the quality of the muscle, which is highlighted above all in an alteration of performance.

The objective of an assessment focused on sarcopenia is to be able to carry out a “tailor-made treatment”.

There is still little data about sarcopenia’s role in patients with malignant hematological diseases. Some data suggest that sarcopenia is associated with lower overall survival in patients with DLBCL treated with modern chemotherapy (R-CHOP or R-mini-CHOP). On the other hand, sarcopenia does not predict overall survival in leukemia patients, so further investigations are needed to analyze the role of muscle loss in different leukemias. Similarly, only one study reported an association between sarcopenia and OS in multiple myeloma.

New research in this field would be desirable.

Key Points:

- Sarcopenia is a geriatric syndrome characterized by a progressive loss of systemic muscle mass plus a decrease in muscle strength or physical function;
- Sarcopenia has a multifactorial pathogenesis: nutritional deficits, physical inactivity, and chronic diseases are the leading causes of sarcopenia;
- Sarcopenia is associated with adverse outcomes such as falls, functional decline, frailty, disability, multiple hospitalizations, and mortality;
- When low muscle mass, low muscle strength, and low physical performance are simultaneously present in a patient, we refer to it as severe sarcopenia;
- When sarcopenia is diagnosed as an obese condition, we have a far more complex syndrome than the sum of the two, called sarcopenic obesity;
- Although sarcopenia and cachexia share standard features, they are two distinct conditions for which a differential diagnosis is needed to best manage our patients;
- In oncology, sarcopenia is associated with an increased risk of treatment toxicity, postoperative complications, sensitivity to anti-blastic treatments, and a higher mortality rate;
- The most effective intervention for reversing sarcopenia is physical exercise combined with nutrition;
- In patients with hematologic malignancy, low muscle mass is associated with adverse outcomes and is a predictor of overall survival and non-relapse mortality.

## Figures and Tables

**Table 1:** Sarcopenia’s measurement tools.

Strength Measurement				
Tool		Cut Off	PROs	Cons
Grip strength (kg)	Assessment of hand grip strength using a dynamometer.	<27 men; <16 women	low-cost easy to use	unreliable in patients with hand disability or cognitive decline difficult to administer in patients with dementia
Chair stand test (5 rises) (s)	Amount of time needed for a patient to rise five times from a seated position without using their arms	>15 s	inexpensive measures both strength and endurance	needs relative space in the room difficult to administer in patients with dementia
Performance measurement				
Tool		Cut Off	PROs	Cons
Gait speed (m/s) 4-m usual walking speed test	The time needed for a patient to walk, at his or her usual speed, a linear path of 4 m	≤0.8	quick easy to perform inexpensive powerful predictor	needs space
SPPB (point/12)	a test consisting of 3 instruments, each assigned a maximum score of 4: (1) gait speed, (2) chair stand test, and (3) balance test. The balance test consists of measuring how long the patient can maintain specific positions in standing	≤8	consists of 3 individually readable tools inexpensive measures both strength and endurance	time-consuming (at least 10 min to administer) difficult to administer in patients with dementia needs space
Timed Up and Go test (s) TUG	The tool consists of getting up from a chair, walking for 3 m, turning around, walking back, and sitting down again	≥20 s	inexpensive predict mortality	difficult to administer in patients with dementia needs space
400 m walking test	Participants are asked to complete 20 laps of 20 m, each lap as fast as possible, and are allowed up to two rest stops during the test	Non-completion or ≥6 min for completion	inexpensive predict mortality	needs space time-consuming
Muscle mass measurement: appendicular skeletal muscle index more than two SDs below that typical of healthy adults (i.e., 5·45 kg/m^2^ for women and 7·26 kg/m^2^ for men)				
Tool		Cut Off	PROs	Cons
Dual-energy X-ray absorptiometry (DXA)	Whole-body skeletal muscle mass (SMM) or appendicular skeletal muscle mass (ASM), both adjusted for height^2^, weight or BMI	Skeletal Muscle Mass Index (Appendicular skeletal muscle mass/height^2^) <7 men kg/m^2^; <5.5 women kg/m^2^	precise measures whole muscle mass	expensive not common in most healthcare realities not portable for use in the community
Bioelectrical impedance analysis (BIA)	Whole-body skeletal muscle mass (SMM) BIA equation of Janssen et al.: skeletal muscle mass (kg) [(height^2^/BIA-resistance 0.401) (gender 3.825) (age 0.071)] 5.102	SM/height^2^: Men: 8.87 kg/m^2^ Women: 6.42 kg/m^2^	low cost portable	measures muscle mass indirectly based on whole-body electrical conductivity measurements can be influenced by hydration status
CT or MRI	Lumbar muscle cross-sectional L3 area Mid-thigh muscle area psoas muscle		already available in cancer patients without the need to do additional examinations predictor of mortality Lumbar muscle across sectional L3 correlates better with whole muscle mass	requires highly-trained personnel to use the equipment and to contour muscle areas time-consuming when there is no auto-contouring program Mid-thigh muscle area and psoas muscle area are less correlated with whole muscle mass

**Table 2:** Comparison between sarcopenia and cachexia.

	Sarcopenia	Cachexia
Weight	↓ or = or ↑↑	↓↓
Lean Tissue	↓↓	↓↓
Fat Tissue	= or ↑	↓
Appetite	=	↓
Cortisol	=	↑
Inflammation	= or ↑	↑↑↑
Pathway	It does not lead to cachexia	May lead to sarcopenia

Refs. [31,32,33,34,35,36,37,38]. ↓: reduction; ↓↓: reduction (moderate); =: unchanged; ↑: increase (low); ↑↑: increase (moderate); ↑↑↑: increase (high).

**Table 3:** Frequent Underlying Causes of Sarcopenia [1].

Nutritional
Low protein intake Low energy intake Micronutrient deficiency Malabsorption and other gastrointestinal conditions Anorexia
Inactivity
Bed rest, immobility, deconditioning Low activity, sedentary lifestyle
Disease
Bone and joint diseases Cardiorespiratory disorders, including chronic heart failure and chronic obstructive pulmonary disease Metabolic disorders (particularly diabetes) Endocrine diseases (particularly androgen deprivation) Neurological disorders Cancer Liver and kidney disorders
Iatrogenic
Hospital admission Drug-related

## Data Availability

No data produced.
